# A Real-Time Monitoring Method for Selective Laser Melting of TA1 Materials Based on Radiation Detection of a Molten Pool

**DOI:** 10.3390/mi15050570

**Published:** 2024-04-26

**Authors:** Tao Zhou, Wei Huang, Congyan Chen

**Affiliations:** 1 School of Automation, Southeast University, Nanjing 210096, China; 220211830@seu.edu.cn (T.Z.); 220211823@seu.edu.cn (W.H.); 2 Shenzhen Research Institute, Southeast University, Shenzhen 518000, China

**Keywords:** selective laser melting, radiation detection, molten pool, orthogonal experiment, numerical simulation

## Abstract

Selective laser melting (SLM) technology is a promising additive manufacturing technology. However, due to the numerous influencing factors in this complex process, a reliable real-time method is needed to monitor the forming process of SLM. The molten pool is the smallest forming unit in the SLM process, the consistency of which can effectively reflect the quality of the printing process. By using a coaxial optical path structure and a compound amplifier circuit, high-speed acquisition of molten pool radiation can be realized. Next, single factor analysis and orthogonal experimentation were used to investigate the influence levels of key process parameters on the radiation of molten pool. In addition, numerical simulation was carried out with the same parameter setting schemes, the results of which are consistent with those in radiation detection experiments. It is shown that the laser power has the greatest effect on the radiation of the molten pool, while the scanning speed and the hatch spacing have little effect on the radiation. Finally, the positioning experiment involving the small hole structure was carried out, and the experimental results showed that the device could accurately locate the position coordinates of the given hole structure.

## 1. Introduction

In the SLM forming process, the stability of the molten pool can directly reflect the forming quality of the entire printing process. The shape and the size of the molten pool directly determine the quality of the formed part. Because of the fast scanning speed of the SLM process, the small molten pool area, and the complex printing process conditions, clarifying how to achieve fast and accurate detection of the molten pool is the focus of the current research on SLM molten pool detection. According to the different molten pool signals of the detection methods, the current detection methods of molten pool are mainly classified as follows: spectral signal detection, temperature signal detection, and topography signal detection [1].

In the detection of spectral signals, the detection objects include element composition and temperature distribution. Photodiodes and spectrometers are commonly used as sensors. Gu et al. [2] established a set of real-time monitoring systems for laser molten pools; used a mercury lamp for spectral calibration, and grating spectral detection technology for spectral characteristics analysis; and studied the spectral radiation intensity distribution of melt pools during the forming process of nickel–silicon–boron alloy powder under different process parameters. Chen et al. [3] established a spectrum acquisition system using fiber optic spectrometers to study the influence of plasma on laser energy transmission efficiency and forming quality under different process parameters, as well as the relationship between the formation of defects and plasma intensity fluctuations. At present, due to the limited means of detection for laser melt pool spectral signals, the degree of feature analysis is shallow and there is more interference, so the research progress is slow. In terms of temperature detection, the most commonly used methods are non-contact radiation temperature measurements, including monochromatic temperature measurement, colorimetric temperature measurement, and CCD image signal acquisition temperature measurement. Yuan et al. [4] studied the online detection and closed-loop control of molten pool temperatures, combined with the colorimetric temperature measurement principle and photoelectric detection technology, and successfully measured the temperatures and spectral radiation characteristics of molten pools. Sun et al. [5] set up a closed-loop system for the online monitoring and control of molten pool temperature with color CCD; conducted orthogonal experiments by combining colorimetric measurement and PID control; and derived and designed the calculation formula and temperature controller algorithm of molten pool temperature, finally realizing stable control of molten pool temperature and obtaining good forming quality. The measurement of molten pool geometry morphology mainly measures the size (length, width) and area of molten pools. The geometry morphology can reflect the temperature field distribution, powder absorption, and condensation rate during processing, so as to reflect the stability of the molten pool during processing. Thompson and Vandone et al. [6,7] developed a vision-based monitoring system based on beam coaxial imaging to study visible radiation anomalies during laser deposition, which integrates optical cameras and narrowband filters to capture and improve image quality. Mazzoleni et al. [8] established a real-time continuous monitoring system for laser molten pool using CMOS cameras, and studied the physical dynamics of molten pools under modulated or continuous laser emission, including emission shape, spatter ejection, and size change of the molten pools.

In the numerical simulation of SLM, the main research focus is the heat source models of laser heat sources. For the heat sources used in SLM numerical simulations, surface heat sources and body heat sources are mainly used. The surface heat source acts on the surface of the powder layer in the form of heat flux, while the body heat source acts on the inside of the powder layer in the form of heat generation rate [9]. Some numerical scholars regard the surface heat source as a uniform Gaussian surface heat source, and some scholars use the double ellipsoid heat source or ellipsoid heat source to conduct the finite element simulation of SLM, and the simulation results are also consistent with previous experiments [10,11,12,13,14,15]. According to the actual processing process, different scholars have established a heat source model combining the surface heat source and the body heat source [16,17,18].

**Figure 1:**
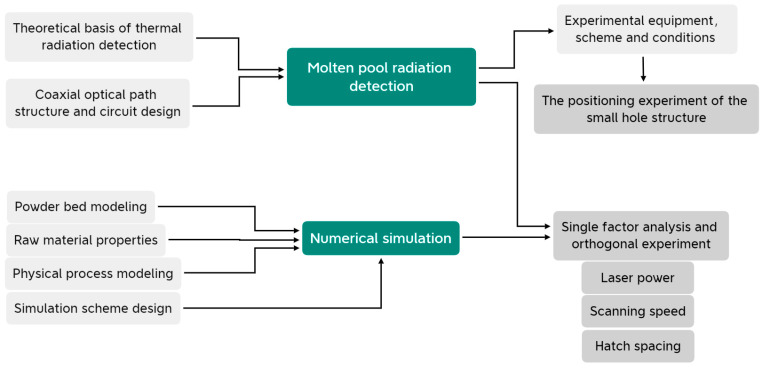
The framework of this research.

The studies mentioned above have made many breakthroughs and solved many key problems in the SLM process. In this study, a set of radiation monitoring systems of molten pools has been designed for SLM. The device uses a photodiode to detect molten pool radiation through a coaxial optical path structure, locks the strongest band of molten pool radiation through a narrowband filter, and then converts the radiation intensity into an analog voltage by using a special amplifier circuit with a composite structure. The voltage is sampled and communicated through an embedded microprocessor to realize online monitoring of SLM molten pool radiation. Figure 1 is the research framework of this study. This research will be based on the self-developed detection method printing experiment, and the numerical simulation method will be used to verify the correctness of the detection results. This study will carry out single-parameter influence experiments and orthogonal experiments with multiple parameter combinations to explore the influences of different process parameters on the radiation of the molten pool during the SLM process. In addition, the localization experiment involving the small hole structure will be carried out to verify that the proposed method can quickly and effectively locate the given small hole structures during the printing process.

## 2. Materials and Methods

### 2.1. Experimental Raw Material

TA1 standard powder (BLT Inc., Xian, China) was used as the experimental raw material. TA1 material has strong antioxidant properties and stable tissue, and the printed workpiece has good mechanical properties and is used in the biomedical industry to create implants with the same excellent mechanical properties as bone. Compared with other alloys, TA1’s elastic modulus is closest to human bone, and it has good biocompatibility without causing adverse reactions in the human body [19,20,21,22]. 

The BT-9300Z laser particle size distributor (Dandong Baxter, Dandong, China) was used to analyze the particle sizes of the experimental powder, and the distribution of powder particle diameters is shown in Table 1. The particle size distribution method is a statistical method. In the particle size distribution method, three special percentages, namely D10, D50s, and D90, are usually selected as characteristics. These three percentages are percentage by volume. Take D10 as an example, which corresponds to a value of 18.94. This means that the proportion of particles with a diameter less than 18.94 µm in the powder material is 10%. 

The physical and thermal properties of the TA1 material involved in the numerical simulation are shown in Table 2.

### 2.2. Theoretical Basis of Thermal Radiation Detection

The temperature measurement mainly includes contact temperature measurement and non-contact temperature measurement. For the molten pool in the SLM process, if the contact temperature measurement is used, it will not only destroy the thermal evolution process of the molten pool itself, but also because the existence time of the molten pool is too short, the temperature cannot be accurately obtained. The use of non-contact, which is mainly based on radiation detection, means we can avoid the above problems.

Before introducing the principle of thermal radiation detection, we first introduce the definition of two variables. Radiant flux and radiant exitance M are defined as follows [24]:

(1) $$\Phi =\frac{dQ}{dt}$$

where Q (J) is the total radiation energy of the whole band and represents the rate of change in the total radiation energy Q of the whole band with time t (s).

(2) $$M=\frac{d\Phi }{dS}=\frac{\partial^{2}Q}{\partial S\partial t}$$

where M (W/m^2^) is the radiation flux emitted by a unit area surface radiation source into a solid angle space. Radiant exitance is a function of wavelength and temperature. According to Planck’s blackbody radiation law, the radiation energy density distribution of an object with temperature T over the wavelength $\left[\lambda ,\lambda +\Delta \lambda \right]$ range is as follows [25]:

(3) $$E\left(\lambda ,T\right)=\frac{8\pi hc}{\lambda^{5}\left(e^{\frac{hc}{kT\lambda }}-1\right)}$$

The radiation exitance at this wavelength and temperature is related to the radiation energy density distribution, as follows:

(4) $$M\left(\lambda ,T\right)=\frac{c}{4}E\left(\lambda ,T\right)=\frac{c}{4}\cdot \frac{8\pi hc}{\lambda^{5}\left(e^{\frac{hc}{kT\lambda }}-1\right)}=\frac{2\pi hc^{2}}{\lambda^{5}\left(e^{\frac{hc}{kT\lambda }}-1\right)}=\frac{C_{1}}{\lambda^{5}\left(e^{\frac{C_{2}}{\lambda T}}-1\right)}$$

In Formulas (3) and (4), h (J·s) represents the Planck constant; c (m/s) represents the speed of light; k (J/K) represents the Boltzmann constant; C_1_ ($W/m^{2}$) represents the first Planck constant, which can be expressed as $C_{1}=2\pi hc^{2}$; C_2_ (K·m) represents the Planck second constant and can be expressed as $C_{2}=\frac{hc}{k}$. 

According to Win’s displacement law, when the temperature is low, the radiation energy is less, and the radiation distribution is mainly concentrated in the infrared band; as the temperature increases, the radiation energy will increase, and the spectral distribution begins to move in the short wave direction. At the same time, Venn’s displacement law also shows that when the temperature of T’s radiation emission reaches the maximum value, it corresponds to a specific wavelength λ m, and the product of the wavelength and temperature is a certain value.

If the wavelength λ of Equation (4) is integrated, the Stefan–Boltzmann law can be obtained. The formula is briefly described below [26]:

(5) $$Q\left(T\right)=\int_{0}^{+\infty }\frac{C_{1}}{\lambda^{5}\left(e^{\frac{C_{2}}{\lambda T}}-1\right)}d\lambda =\frac{C_{1}}{C_{2}^{4}}\Gamma \left(4\right)\sum_{K=1}^{+\infty }\left(\frac{1}{K^{4}}\right)T^{4}=\frac{2\pi^{5}K^{4}}{15c^{2}h^{3}}T^{4}=\sigma T^{4}$$

where σ ($W/(m^{2}\cdot K^{4}$) represents the Stefan–Boltzmann constant. This equation shows that the total radiation energy of the object in the full band is proportional to the fourth cubic energy of its temperature. In practical applications, the radiation energy in a certain wavelength range is usually detected. For an object of temperature T, the radiation energy in the wavelength range $\left(\lambda_{1},\lambda_{2}\right)$ can be expressed as follows:

(6) $$Q_{\Delta \lambda }\left(T\right)=\int_{\lambda_{1}}^{\lambda_{2}}M\left(\lambda ,T\right)d\lambda =\int_{\lambda_{1}}^{\lambda_{2}}\frac{C_{1}}{\lambda^{5}\left(e^{\frac{C_{2}}{\lambda T}}-1\right)}d\lambda \;=\sum_{n=1}^{+\infty }\frac{C_{1}T^{4}}{C_{2}^{4}n^{4}}\left[{\left(\frac{nC_{2}}{\lambda_{2}T}\right)}^{3}+3{\left(\frac{nC_{2}}{\lambda_{2}T}\right)}^{2}+6\left(\frac{nC_{2}}{\lambda_{2}T}\right)+6\right]e^{-\frac{nC_{2}}{\lambda_{2}T}}-\sum_{n=1}^{+\infty }\frac{C_{1}T^{4}}{C_{2}^{4}n^{4}}\left[{\left(\frac{nC_{2}}{\lambda_{1}T}\right)}^{3}+3{\left(\frac{nC_{2}}{\lambda_{1}T}\right)}^{2}+6\left(\frac{nC_{2}}{\lambda_{1}T}\right)+6\right]e^{-\frac{nC_{2}}{\lambda_{1}T}}\;=C_{1}\times 10^{16}\times \left[\sum_{n=1}^{+\infty }\left[\left(\frac{T}{nC_{2}{\lambda_{2}}^{3}}\right)+3\left(\frac{T^{2}}{{{n^{2}C}_{2}}^{2}{\lambda_{2}}^{2}}\right)+6\left(\frac{T^{3}}{{{n^{3}C}_{2}}^{3}\lambda_{2}}\right)+6\left(\frac{T^{3}}{{{n^{4}C}_{2}}^{4}}\right)\right]e^{-\frac{nC_{2}}{\lambda_{2}T}}\right]-C_{1}\times 10^{16}\times \left[\sum_{n=1}^{+\infty }\left[\left(\frac{T}{nC_{2}{\lambda_{1}}^{3}}\right)+3\left(\frac{T^{2}}{{{n^{2}C}_{2}}^{2}{\lambda_{1}}^{2}}\right)+6\left(\frac{T^{3}}{{{n^{3}C}_{2}}^{3}\lambda_{1}}\right)+6\left(\frac{T^{3}}{{{n^{4}C}_{2}}^{4}}\right)\right]e^{-\frac{nC_{2}}{\lambda_{1}T}}\right]$$

As shown in Equation (6), the radiation energy in the wavelength range $\left(\lambda_{1},\lambda_{2}\right)$ is a function of the off interval range and temperature T. This study does not measure the temperature of the molten pool directly, but characterizes the temperature of the molten pool by obtaining the radiant brightness of the molten pool. This is reliable and easy to implement in engineering applications.

### 2.3. Circuit Design and System Structure

**Figure 2:**
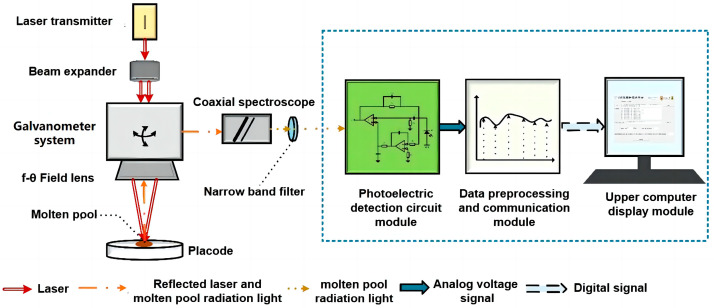
Radiation detection method system block diagram.

The molten pool radiation monitoring system designed in this paper includes a photoelectric detection circuit module, a data preprocessing and communication module, and an online display module of molten pool radiation data. The system block diagram of the detection device is shown in Figure 2. The following will mainly introduce the self-designed and completed photoelectric detection module.

In order to cope with the frequently changing SLM molten pool radiation signal, the photodiode used in the detection circuit needs to have the characteristics of a wide band, high sensitivity, and fast response speed. In this paper, the silicon PIN photodiode s5973-02 from Hamamatsu Photonics (Hamamatsu City, Shizuoka Prefecture, Japan) was selected. The spectral response range of the photodiode is 320–1000 nm.

#### 2.3.1. Compound Amplifier Circuit

Considering that the actual induced output of the photodiode photocurrent signal is below µA level and contains certain noise, a high signal-to-noise ratio amplifier circuit is needed for precise detection. Because the radiation signal of molten pool changes quickly and contains rich spectral information, the amplifier circuit is required to have a better response to high frequency signals. The bandwidth of operational amplifiers for the precise detection of weak signals is often small, and it is difficult to meet the required response rate for radiation detection in molten pools. Broadband and high-speed operational amplifiers often have high low-frequency noise as well as large offset voltage and current, and struggle to detect weak signals. The conventional operational amplifier circuit composed of a single amplifier cannot meet the demand of SLM molten pool radiation detection. In this paper, an amplifier circuit with a compound structure is designed. OP07 and AD8065 operational amplifiers are used together to compromise their respective performance to meet the requirements of detection. 

**Figure 3:**
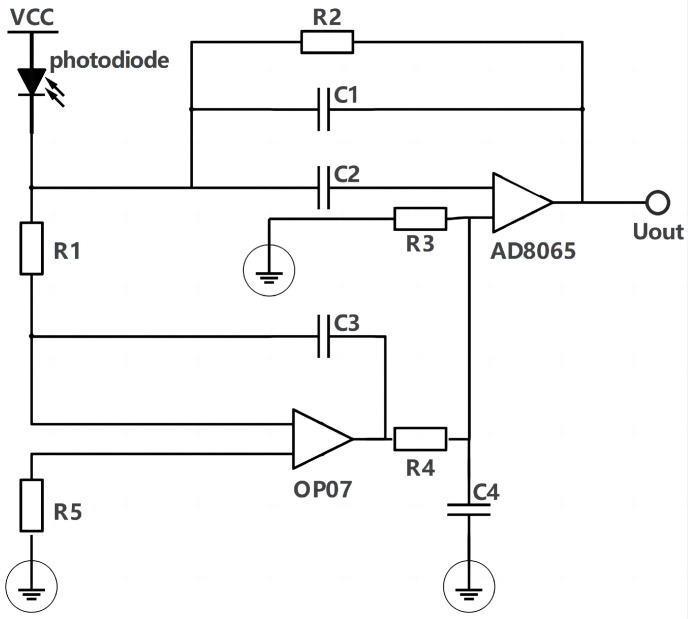
Schematic diagram of compound amplifier circuit.

**Figure 4:**
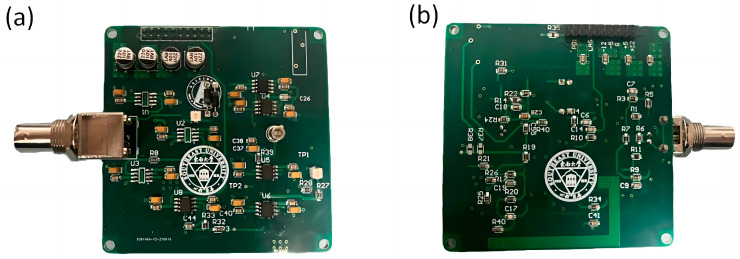
Photoelectric detection circuit physical diagram (**a**) right side, (**b**) reverse side.

As shown in Figure 3, the photoelectric detection pre-circuit is a special circuit structure that combines two types of operational amplifiers, and forms the effect of cross-resistance amplification for the weak current signal output by the PIN photodiode. Figure 4a,b show the display of the photoelectric detection device.

#### 2.3.2. Coaxial Optical Path Structure

**Figure 5:**
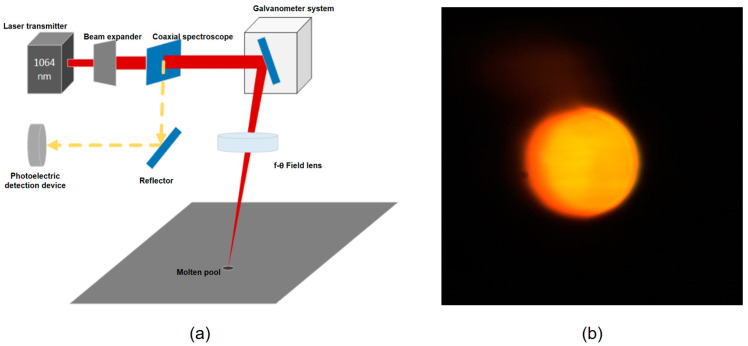
(**a**) Coaxial optical path structure diagram; (**b**) molten pool detected by coaxial optical path.

The optical path system used in SLM process monitoring is usually improved on the basis of the existing optical path system. According to the relative position of the sensor and the laser optical path, the optical path system used for detection can be divided into an off-axis optical path system and a coaxial optical path system. The installation method chosen in this paper is the coaxial optical path system, as shown in Figure 5a. The coaxial optical path system actually observes a local region centered on the current laser position, which is shown in Figure 5b.

The radiant light of the molten pool contains rich spectral information. For different metal materials, a band that can reflect the properties of the material should be selected for radiation detection. The energy level distribution of different elements is different, and the line spectrum contains the characteristic information of various atoms in the material, and the corresponding wavelength and light intensity distribution also reflect the properties of the material [27,28]. For a given material, there may be multiple lines of the spectrum. Considering that the radiant light signal of the molten pool itself is very weak, and the narrowband filter has an attenuation effect on the radiant light, the line spectrum with the greatest light intensity will be detected during practical applications. 

### 2.4. Numerical Simulation

**Figure 6:**
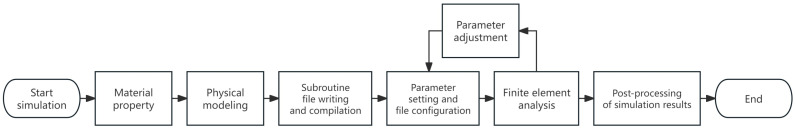
Flow chart of numerical simulation process.

The numerical simulation part of this study was mainly used to check the correctness of the detection results of the radiation detection device. The numerical simulation section mainly refers to a part of the existing simulation methods and schemes. The following mainly introduces the modeling and simulation process of the simulation. The simulation software used in this study is EDEM (version 2.7.0) and Flow3d (version 11.1). Figure 6 shows the overall flow diagram of the simulation.

#### 2.4.1. Powder Bed Modeling

**Figure 7:**
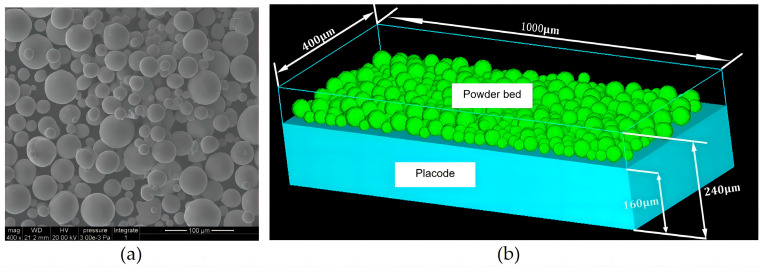
(**a**) Electron microscope observation of powder bed; (**b**) particle bed modeling diagram.

The powder bed of the selective laser melting process is a discrete particle model with certain distribution rules. In this study, the discrete element method (DEM) [29] was used to establish a powder bed model approximating the actual particle distribution. The modeling data of the powder bed is derived from the actual powder data in Table 1, which brings the simulation process closer to the actual printing process. Figure 7 shows the actual electron microscope observation diagram of the powder bed and the modeling diagram of the powder bed. By comparison, it can be found that the particle distributions of the two are very similar. The powder bed model established by simulation in this paper is a 1000 × 400 × 240 μm powder bed, which is a single-layer powder bed model.

#### 2.4.2. Physical Process Modeling

The boundary conditions of the SLM simulation process need to consider heat exchange, heat radiation, and heat convection at the same time. The boundary conditions can be set as follows [30]:

(7) $$q=-k\frac{\partial T}{\partial z}+h\left(T-T_{0}\right)+\sigma \varepsilon \left(T^{4}-T_{0}^{4}\right)$$

where h (W/(m^2^°C)) is the heat convection coefficient, σ is the Stefan–Boltzmann constant, T (°C) is the temperature, and ε is the radiation coefficient of the TA1 powder to the laser.

The laser used in this study is a single-mode fiber laser with a wavelength of 1064 nm. The mathematical expression of this heat source is as follows [31]:

(8) $$Q=\frac{2AP}{\pi r_{0}^{2}d}exp\left(-2\frac{r^{2}}{r_{0}^{2}}\right)\cdot exp\left(-\frac{z}{d}\right)$$

where P (W) is the laser power; $r_{0}$ (m) is the effective laser beam radius when the energy density of the center of the laser spot is reduced to 1/e^2^; r (m) is the radial distance from a point on the surface of the powder bed to the center of the laser spot; d (m) is the optical penetration depth; and A is the absorption rate of the material to the laser.

The above is mainly to model the physical changes of the SLM forming process, and in the simulation process, these physical processes are realized through secondary development by way of subroutines.

## 3. Results

The printing equipment used in this research is a TI150 SLM metal printer from Nanjing Profeta Intelligent Technology Company. The setting ranges of the process parameters below are based on the recommendations of the equipment manufacturer and the analysis of previous experimental results.

### 3.1. Single Factor Analysis

#### 3.1.1. Laser Power

**Figure 8:**
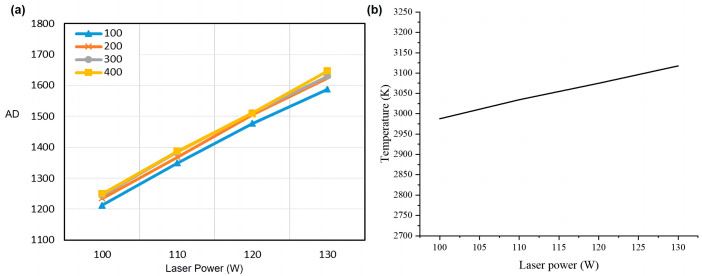
(**a**) The influence of laser power on the radiation signal (AD) of the molten pool; (**b**) the numerical simulation result.

In order to study the specific effect of laser power on molten pool radiation during SLM, four 8 mm × 8 mm squares were printed experimentally, with a total of 500 layers. The laser power of the four blocks was 100 W, 110 W, 120 W, and 130 W, layer thickness was 30 µm, scanning speed was 1000 mm/s, hatch spacing was 0.08 mm, and the scanning mode was unidirectional scanning. For these four test pieces, the signals of all fuses at 100~500 layers (100 layers apart) were selected for each test piece, and their average values were calculated as shown in Figure 8a. Numerical simulation was carried out under the same experimental parameter setting conditions, and the simulation results are shown in Figure 8b. The value of the ordinate in Figure 8b is the average temperature in the center area of the molten pool.

As can be seen from Figure 8a, the radiation signal of the molten pool gradually increases with the increase in laser power, which is because the higher the laser power, the more energy is absorbed by the TA1 powder, and as such the temperature of the molten pool will also rise. The numerical simulation results in Figure 8b are consistent with the results of the radiation detection of the molten pool, both of which increase linearly with the increase in laser power. And with the increase in printing layers, the radiation signal also grows; this is because the metal material undergoes rapid melting and cooling solidification, and so the heat does not completely subside; after layer accumulation, more and more heat accumulates in the forming part, and the molten pool radiation is also increased.

#### 3.1.2. Scanning Speed

**Figure 9:**
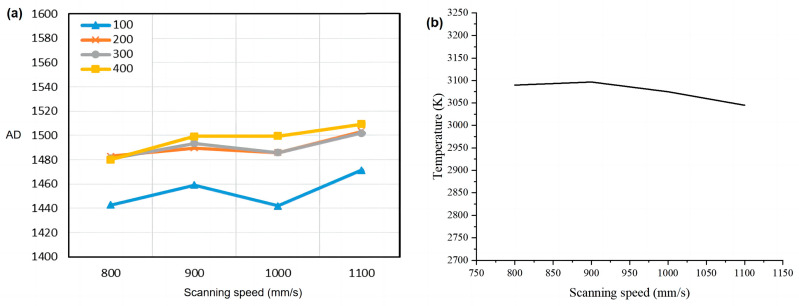
(**a**) The influence of scanning speed on the radiation signal (AD) of the molten pool; (**b**) the numerical simulation result.

The experiment printed four 8 mm × 8 mm squares with a total of 500 layers. The scanning speed of the four squares was 800 mm/s, 900 mm/s, 1000 mm/s, and 1100 mm/s, layer thickness was 30 µm, laser power was 120 W, hatch spacing was 0.08 mm, and the scanning mode was unidirectional scanning. For these four test pieces, the signals of all fuses at the height of the same layer were selected for each test piece, and their average values were calculated. A curve diagram of the radiation signal and scanning speed of the molten pool can be drawn, as shown in Figure 9a. Numerical simulation was carried out under the same experimental parameter setting conditions, and the simulation results are shown in Figure 9b. The value of the ordinate in Figure 9b is the average temperature in the center area of the molten pool.

As can be seen from Figure 9a,b, when the laser scanning speed is 800 mm/s, the temperature and radiation of the molten pool are both low. Because the size of the molten pool is large in the case of low scanning speed, the liquid metal in the molten pool can rapidly absorb heat and transfer it to the surrounding areas, so the temperature of the molten pool’s center is not high. When the scanning speed is increased to 900 mm/s, the time taken for laser action is reduced, the heat conduction capacity of the molten pool is reduced, and the temperature of the molten pool is increased. With the increase in scanning speed, the laser input takes the dominant position, and the molten pool’s temperature decreases with the increase in scanning speed. The molten pool radiation result is inconsistent with the result of the molten pool temperature simulation after 1000 mm/s. At this time, the increase in the molten pool radiation detection value is mainly because the number of molten pools in the coaxial field of view will increase with the increase in scanning speed. Therefore, when the speed is higher, the molten pool radiation detection device detects the superposition value of multiple molten pools’ radiation. 

#### 3.1.3. Hatch Spacing

**Figure 10:**
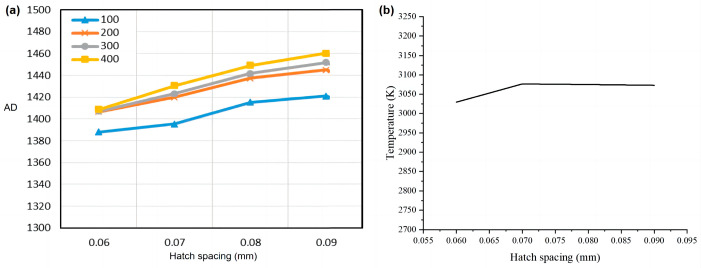
(**a**) The influence of hatch spacing on the radiation signal (AD) of the molten pool; (**b**) the numerical simulation result.

The exploration of scanning spacing is mainly to ensure that the temperature field formed by the laser spot can cover the gap between the two adjacent scanning paths, so that all the regional powder can be melted. During the experiment, we printed four 8 mm × 8 mm squares with a total of 500 layers. The hatch spacing of the four squares was 0.06 mm, 0.07 mm, 0.09 mm, and 0.09 mm, layer thickness was 30 µm, laser power was 120 W, scanning speed was 1000 mm/s, and the scanning mode was unidirectional scanning. For these four test pieces, the signals of all fuses at the height of the same layer were selected for each test piece, and their average values were calculated. A curve diagram of the radiation signal and scanning speed of the molten pool can be drawn, as shown in Figure 10a. Numerical simulation was carried out under the same experimental parameter setting conditions, and the simulation results are shown in Figure 10b. The value of the ordinate in Figure 10b is the average temperature in the center area of the molten pool.

It can be seen from Figure 10a,b that the hatch spacing has little influence on the signal radiation and the temperature of the molten pool. The increase in hatch spacing means that the lap rate between the molten pool and the adjacent fuse decreases, and the amount of powder in the fuse gap increases. The thermal conductivity of the powder is lower than that of the liquid molten pool, so when the hatch spacing is larger, the fluidity of the molten pool is reduced, the heat transferred by the molten pool to the adjacent molten channel is reduced, and the energy radiated by itself will increase.

### 3.2. Orthogonal Experiment

#### 3.2.1. Orthogonal Experimental Design

Laser power, scanning speed, and hatch spacing are the three main parameters that affect the SLM forming process. In this experiment, the layer thickness is set to 30 µm, and the three process parameters of laser power, scanning speed, and hatch spacing are tested and analyzed by the orthogonal experimental design method. The main problem of the comprehensive test is that the number of horizontal combinations is large and the workload is large, so the orthogonal experiment design method has been chosen in this study. The basic feature of orthogonal experiment design is to replace a full experiment with a partial experiment, and to understand the situation of the full experiment through the analysis of partial test results. 

The orthogonal test method uses the test scheme of the orthogonal table to achieve the conditions of the comprehensive test through the analysis of partial test results. Table 3 shows the parameter setting range of the experiment in this paper as well as the number of the corresponding parameters.

The parameter design and experiment sequence number of the orthogonal experiment are shown in Table 4.

#### 3.2.2. Orthogonal Experimental Results

The results of the orthogonal experiment are shown in Table 5. In Table 5, $k_{i}$ indicates the average value of all test values whose serial number is i in the column where the corresponding parameter resides. Detailed parameter settings are shown in Table 4. For example, $k_{1}$ for power P is the average value of the detected values for experiment numbers 1 to 4 (Table 4). Each value in the table reflects the average value under the corresponding setting for this parameter. The range R (the difference between the maximum and minimum values) reflects the variation in the radiation of the molten pool when the level of the parameter in this column fluctuates. The greater the range R, the greater the influence of this factor on the radiation of the molten pool. The primary and secondary order of factors can be judged according to range R. The values in the table are AD values without units.

From the range analysis of the results in Table 5, it can be seen that $R_{P}=467.30>\;R_{V}=103.04>R_{H}=114.97,$ which means that the laser power has the greatest influence on the radiation of the molten pool, while the scanning speed and the scanning distance have little influence on the radiation of the molten pool. 

At the same time, the numerical simulation was carried out according to the parameter setting scheme in Table 4, and the results were analyzed as shown in Table 6. The numerical calculation methods in Table 6 are the same as in Table 5. The values in the table all represent the temperature (K).

As shown in Table 6, $R_{P}=130.0>R_{V}=51.6>R_{H}=47.2,$ which is consistent with the experimental results of radiation detection in the molten pool. The results show that the detection results of the radiation monitoring device are in agreement with the simulation results of the molten pool temperature, proving the correctness of the detection device’s detection results. Of course, since the result of radiation detection is an AD value with adjustable gain, further correlation to the temperature of the molten pool needs to be calibrated in future works.

### 3.3. Positioning Experiment

#### 3.3.1. Detection and Positioning of Concave Structure

**Figure 11:**
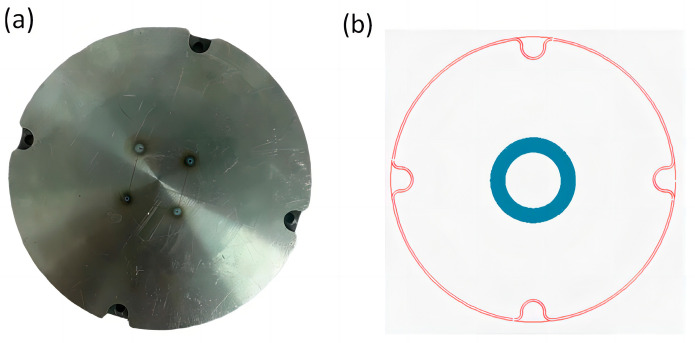
(**a**) Perforated substrate; (**b**) print document design.

In order to test the effectiveness of the molten pool radiation monitoring system, as shown in Figure 11a,b, four circular holes with a diameter of 0.5 mm were first punched in the SLM printing substrate, and then the four circular holes were covered through printing. The function of the monitoring system was judged by whether the molten pool radiation fluctuated abnormally when the laser was printing on the round holes and the substrate.

#### 3.3.2. Detection Result

**Figure 12:**
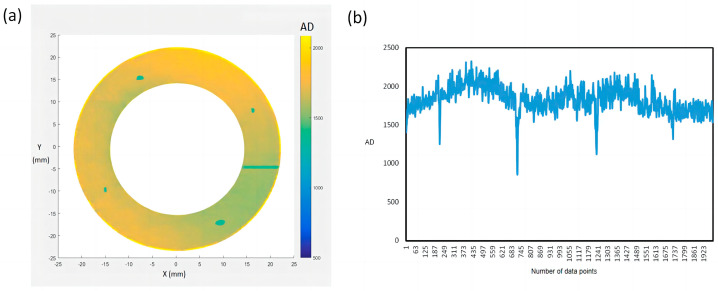
(**a**) Two-dimensional and (**b**) one-dimensional data of molten pool radiation.

The main purpose of the molten pool radiation detection device developed in this research is to realize defect detection during the SLM process, and then realize the closed-loop control. The detection results of the detection device were verified as correct by comparing the numerical simulation results with the radiation detection results. The aim of the following is to verify the effectiveness of this method from the detection function. Figure 12 shows the two-dimensional (a) and one-dimensional (b) data of molten pool radiation obtained from the positioning experiment. 

It can be clearly seen from Figure 12a,b that the radiation value of the molten pool at the four holes is low, because the holes are made of TA1 metal powder after the powder is laid, while the substrate is made of 316L stainless steel. The thermal physical parameters of the two materials are different, resulting in significant changes in the radiation value of the molten pool. In Figure 12a, there is a line with lower radiation, which is the starting and ending line of each fuse, and the laser has lower power at the start and stop points, resulting in less radiation from the molten pool. The experimental results show that the monitoring system can effectively detect the changes in molten pool radiation and accurately locate the specific coordinates of the molten pool. The experimental results show that the molten pool radiation monitoring system can locate the actual molten pool coordinates by distinguishing the molten pool radiation caused by abnormal fluctuations on the surfaces of the parts with small holes. The spatial resolution of the collected data points is smaller than the geometric scale of two molten pools. The positioning accuracy can reach 100–150 µm.

## 4. Discussion and Conclusions

In this paper, a real-time detection method based on radiation detection is proposed for molten pools produced during the selective laser melting process. In order to verify the results of this detection method, a print experiment and numerical simulation were carried out simultaneously. Firstly, single factor analysis and an orthogonal experiment were carried out, which concluded that the results of the radiation detection experiment are consistent with the results of the numerical simulation. They also showed that among the three process parameters of laser power, scanning speed, and hatch spacing, the influence level of laser power on molten pool radiation is the highest, while the influences of scanning speed and hatch spacing are lower. Then, the given holes positioning experiment was carried out. The method reported in this paper can effectively detect the coordinates of given small holes, and the spatial resolution can reach 100–150 µm. Compared with the work done by other scholars in the past, the main advantage of the method designed and completed in this paper is that the response speed is fast and changes in the molten pool’s state can be detected in real time. At the same time, this paper makes a joint analysis and comparison between the method of numerical simulation and the results of radiation detection. Whether it is single-factor experiment or an orthogonal experiment, the detection results of the device are highly consistent with the results of numerical simulation.

Limited by experimental conditions and experimental equipment, there are still many shortcomings in this paper. For example, the measured molten pool radiation could not be calibrated with the actual temperature, and the experimental test pieces could not be tested for forming quality, such as mechanical properties. Further work will be carried out to improve this study.

During the process of selective laser melting, the formation and decay of molten pools are very fast, which makes it difficult to detect the temperature of molten pools in real time. A photoelectric detection method based on a coaxial optical path, which has both a fast response time and high spatial resolution, is proposed in this paper. Real-time and closed-loop control of the SLM process can be realized in the future based on this detection method, which will effectively improve the forming quality of the SLM technology. In addition, the proposed method can also realize the printing quality monitoring of fine structures, which is difficult to do with conventional camera monitoring, because the monitoring accuracy and response time of the camera cannot keep up with the speed of the printing work. Of course, the fusion and use of multi-sensors is the current trend. In the next stage, this study will explore the combined use of multiple monitoring methods, such as industrial cameras, ultrasonic monitoring, X-ray, etc., and realize the fusion of information from multi-sensors on multiple levels such as data, characteristics, and decision-making, so as to realize a fast and reliable SLM molten pool monitoring system.

## Figures and Tables

**Table 1:** Particle diameter distribution of TA1 material.

Proportion (%)	Particle Diameter (μm)
D10	18.94
D50	33.37
D90	58.08

**Table 2:** Physical and thermal properties of TA1 material [23].

Properties	Value
Density	4540 kg/m^3^
Melting point	1667 °C
Latent heat of fusion	2.95 × 10^5^ J/kg
Latent heat of evaporation	8.878 × 10^6^ J/kg
Surface tension coefficient	1.525 N/m

**Table 3:** Parameter range setting table.

	Laser Power P (W)	Scanning Speed V (mm/s)	Hatch Spacing H (mm)
1	100	800	0.06
2	110	900	0.07
3	120	1000	0.08
4	130	1100	0.09

**Table 4:** Orthogonal experimental design table.

	Factors			Laser Power (W)	Scanning Speed (mm/s)	Hatch Spacing (mm)
1	1	1	1	100	800	0.06
2	1	2	2	100	900	0.07
3	1	3	3	100	1000	0.08
4	1	4	4	100	1100	0.09
5	2	1	2	110	800	0.07
6	2	2	3	110	900	0.08
7	2	3	4	110	1000	0.09
8	2	4	1	110	1100	0.06
9	3	1	3	120	800	0.08
10	3	2	4	120	900	0.09
11	3	3	2	120	1000	0.07
12	3	4	1	120	1100	0.06
13	4	1	4	130	800	0.09
14	4	2	3	130	900	0.08
15	4	3	1	130	1000	0.06
16	4	4	2	130	1100	0.07

**Table 5:** Table showing range analysis of orthogonal experimental results.

Index	P	V	H
k_1_	1044.10	1403.46	1389.00
k_2_	1092.70	1300.42	1358.75
k_3_	1312.33	1351.20	1274.03
k_4_	1497.21	1320.05	1329.17
R	467.30	103.04	114.97
Sort	P > V > H		

**Table 6:** Table showing range analysis of numerical simulation results.

Index	P	V	H
k_1_	2987.6	3089.7	3029.3
k_2_	3034.4	3096.5	3076.5
k_3_	3074.9	3074.9	3074.9
k_4_	3117.6	3044.9	3073.1
R	130.0	51.6	47.2
Sort	P > V > H		

## Data Availability

Data are contained within the article.
